# Epidemiology and Response to the COVID-19 Pandemic in Kerala, India, 2020–2021: A Cross-Sectional Study

**DOI:** 10.3390/tropicalmed7060105

**Published:** 2022-06-14

**Authors:** Ajan Maheswaran Jaya, Anthony D. Harries, Anisur Rahman, Mohammed Khogali, Palanivel Chinnakali, Lakshmi Geetha Gopalakrishnan, Mahesh Narayana Pillai

**Affiliations:** 1 Directorate of Health Services, Thiruvananthapuram 695101, India; gglakshmi84@gmail.com (L.G.G.); nmahesh83@gmail.com (M.N.P.); 2 Centre for Operational Research, International Union against Tuberculosis and Lung Disease (The Union), 75001 Paris, France; adharries@theunion.org; 3 Department of Clinical Research, Faculty of Infectious and Tropical Diseases, London School of Hygiene and Tropical Medicine, London WC1E7HT, UK; 4 World Health Organization (WHO), Country Office, New Delhi 110029, India; rahmanan@who.int; 5 Special Programme for Research and Training in Tropical Diseases (TDR), World Health Organization, 1211 Geneva, Switzerland; khogalim@who.int; 6 Department of Preventive and Social Medicine, Jawaharlal Institute of Postgraduate Medical Education and Research, Puducherry 605006, India; palaniccm@gmail.com

**Keywords:** COVID-19, India, Kerala, COVID-19 case notifications, COVID-19 deaths, COVID-19 case fatality, COVID-19 bed occupancy

## Abstract

Kerala, a southern state in India, experienced a slightly different COVID-19 pandemic than the rest of India. Using data from daily COVID-19 bulletins and two other Kerala health information systems, this study reported on epidemiological characteristics and response measures of the COVID-19 pandemic between January 2020 and December 2021. After the first six months, Kerala experienced three distinct phases, with COVID-19 cases peaking in October 2020, May 2021, and August 2021. This contrasts with India, which experienced two main peaks in September 2020 and May 2021. The demographic profile of cases aligned with the national profile except for a slight increase of COVID-19 in persons aged ≥60 years. Monthly COVID-19 deaths increased dramatically from May 2021 onwards in line with case numbers but also reflecting changes in definitions of COVID-19 deaths. Case fatality for the two years was significantly higher in males than females, increased with increasing age groups, and varied between districts (*p* < 0.001). Trends in bed occupancy in field hospitals, hospitals for severe disease, intensive care units, and mechanical ventilation mirrored the different phases of the pandemic. The monitoring system in Kerala allowed certain aspects of the pandemic to be mapped, but it would benefit from further strengthening.

## 1. Introduction

The disease caused by SARS-CoV-2 known as coronavirus disease 2019 (COVID-19) was first reported internationally as a cluster of viral pneumonia cases in Wuhan city, Hubei province, China, in December 2019, and it later spread to countries worldwide [1]. On 30 January 2020, the WHO declared that the outbreak constituted a Public Health Emergency of International Concern (PHEIC), and later in March 2020 it was declared a pandemic. As of the middle of March 2022, there were 455 million confirmed cases and over 6 million deaths across the globe [2].

Kerala, a southern state of India, reported India’s first COVID-19 case on 30 January 2020, in Thrissur district. Since then, cases of COVID-19 have been widely reported across all Indian states. By early March 2020, Kerala state had the highest reported number of active cases in the country, mainly due to cases imported from other countries and states within India. As the number of COVID-19 cases began to increase, the Government of India imposed a complete lockdown starting on 24 March 2020. During the early stage of the pandemic, comprising the pre-lockdown period in India, COVID-19 cases were mainly reported by people with recent international travel history. This was a result of the testing strategy being targeted to this group, and this occurred across all states, including Kerala. From July 2020 onwards, however, the testing strategy in Kerala broadened to include patients with influenza-like illness, severe acute respiratory illness, symptomatic front-line health workers, and so on.

Kerala, a densely populated state in the south of India, was highly vulnerable to rapid transmission and associated morbidity and mortality from the COVID-19 disease. This was due to a number of factors that included a high population density, a large proportion of elderly people, a high prevalence of cardiovascular and non-communicable diseases, and a large inflow of people from other countries. Following the nationwide lockdown from midnight on 24 March 2020, that restricted people’s entry from outside the state, the COVID-19 curve flattened by the third week of April 2020. New cases dropped to near zero by the first week of May, with no evidence of community transmission. In June 2020, however, COVID-19 cases started to increase again.

The increase in cases in Kerala has been different from the rest of India during different time periods. The socio-demographic characteristics of its population, the public health reporting system along with measures adopted by the state during the various phases of the epidemic deserve scrutiny to understand if there are any lessons to be learned that may help to tackle the current pandemic in India and other countries.

The aim of the study, therefore, was to report on the epidemiology of COVID-19 and the control measures implemented in Kerala, India, between January 2020 and December 2021. Specific objectives were to describe: (i) the number of confirmed cases of COVID-19 and the public health responses in Kerala over the two years compared with the trends seen in India; (ii) the demographic characteristics and geographical distribution of cases in Kerala; (iii) the trends in deaths and overall case-fatalities in relation to age, gender, and district; and (iv) hospital bed occupancy rates, including those in intensive care units and with mechanical ventilation.

## 2. Materials and Methods

### 2.1. Study Design

This was a cross-sectional study using surveillance data on COVID-19 in Kerala.

### 2.2. General Setting

Kerala is a state in the southern part of India, with a literacy rate of 94% and a high human development index. The state has a total area of 38,863 sq. km and a 33.4 million population as per the national census 2011 [3]. The per capita Gross State Domestic Product of Kerala was estimated at $3,300 (USD) from 2019 to 2020 [4]. There are 14 districts in Kerala.

Kerala’s preparedness to deal with COVID-19 was evident from the guidelines issued by the state on 26 January 2020, i.e., much earlier than the first case detected in India. The early phase of the pandemic mandated the tracing and isolation of persons coming into contact with infected individuals, thereby preventing or at least limiting the spread of the virus within the community. This public health strategy was informed through the effective containment of the Nipah virus in 2018, with measures that included: contact tracing by rapid response team members, daily review meetings with intersectoral coordination, political commitment, and awareness campaigns against fake news and psychological support to the bereaved and anxious contacts. The public health strategy was further strengthened with the successful ‘Break the Chain’ campaign that urged people to partner in these efforts through self-protection, hygiene, and social distancing [5,6,7,8,9]. The campaign was underpinned by enforcing the disaster management act which mandated the payment of fines for those not adhering to the prescribed measures.

As the COVID-19 pandemic progressed, the information given to the public was continuously modified through various media channels, and the campaign of “Break the Chain” evolved with public health messages repeatedly reinforced [10]. Daily updates regarding the COVID-19 scenario in the state were published by the government of Kerala dashboard (https://dashboard.kerala.gov.in/covid/, accessed on 10 October 2020). Details regarding occupancy in hospitals, bed availability, and other data were published in the COVID-19 Jagratha portal (https://covid19jagratha.kerala.nic.in/, accessed on 10 October 2020). The Jagratha portal also enabled travelers from outside the state and the country to be registered and given permits to enter the state. Both these dashboards were accessible in the public domain.

The main reasons for limiting the spread of the disease were efficient contact tracing, aggressive testing, and effective isolation of those found to be infected. Rapid response team members at the local government level and staff of health facilities at the primary care level were provided with information on new COVID-19 cases on a day-to-day basis through the Jagratha portal. Details of contacts in these cases were gathered through telephone calls and house visits. Patient travel routes were also published in the initial stages of the pandemic so that those in close contact could inform the health authorities, but as cases increased this practice had to be discontinued due to an overburden of work. COVID-19 testing laboratories and specialized coronavirus care centers were developed and scaled up. Scientific laboratories in Kerala were made available and there was a widespread distribution of low-cost COVID-19 testing kits and personal protective equipment, which have continued to be manufactured on a large scale by private companies locally.

Using the five components of trace, quarantine, test, isolate and treat, the state was able initially to contain the spread of infection and maintain the COVID-19 case fatality rate to less than 1%, well below the national average.

### 2.3. Study Population and Study Period

The study included all cases of confirmed COVID-19 in Kerala state, reported from 30 January 2020 to 31 December 2021.

### 2.4. COVID-19 Case Management

Case identification is carried out by the health staff of each district, which includes the District Surveillance Officer and their teams, the Integrated Disease Surveillance Programme (IDSP) team, and the urban health authorities of the major cities. Reporting of Influenza-Like Illness (ILI) and Severe Acute Respiratory Illness (SARI) cases has always been mandated in government hospitals but has also been made mandatory for private health care providers. The contacts of COVID-19-positive people who are in isolation are monitored. The contacts are identified through various measures as described earlier, and they are monitored by rapid response team members and field staff through telephone calls and house visits. These are coordinated at the district level by the district program management group, support unit, and state control room, who can bring in help from the police if required.

With respect to domestic and international travel, the state has a policy of quarantining and subsequently testing all travelers. Most of the travelers have come from COVID-19-affected countries or states within India. During the initial phase of the pandemic, upon arrival in the state, travelers were immediately subject to testing. If they tested positive, they were hospitalized. If they tested negative, they were quarantined for 14 days with a repeat test being carried out after the 7th day of quarantine. From October 2021 onwards, the quarantine policy changed, and the total quarantine period has now been reduced to seven days.

### 2.5. COVID-19 Case Definitions

The following case definitions were used in this study to report on COVID-19 cases.

#### 2.5.1. Confirmed Case of COVID-19

A person with laboratory confirmation of COVID-19 by real-time reverse transcriptase-polymerase chain reaction (RT-PCR), cartridge-based nucleic acid amplification test (CBNAAT), or rapid diagnostic antigen testing, irrespective of clinical signs and symptoms in Kerala [11].

#### 2.5.2. COVID-19 Death

Death due to COVID-19 was defined for surveillance purposes as a death resulting from a clinically compatible illness in a confirmed COVID-19 case unless there was a clear alternative cause of death that could not be related to COVID-19 disease (e.g., trauma), with no period of complete recovery from COVID-19 between illness and death. The definition changed in September 2021 when a COVID-19 death was defined as occurring within 30 days of being tested positive. From October 2021, families could also appeal previously notified causes of death so that missing COVID-19 deaths in the registers could be retrospectively included [12].

### 2.6. Study Variables

Data variables for the study included: date of notification of confirmed cases of COVID-19 in Kerala; demographic factors (age, gender district of residence); district population; death; average monthly bed occupancy of COVID-19 positive cases for field hospitals (which included Domiciliary care centers, COVID first-line treatment centers, and COVID second-line treatment centers), hospitals for more severe cases, intensive care units (ICU) and for mechanical ventilation. Sources of data were: (i) the daily COVID-19 bulletins published on the website of the Department of Health and Family Welfare, Government of Kerala; (ii) reports from the Kerala health data box—this is a data dissemination link in the Department of Health and Family Welfare website, which is managed by the department with monthly updates available to the general public; and (iii) the Kerala health dashboard—this is the website of the Directorate of Health Services, Kerala, with daily updates which are available to the general public [8,13,14]. Case notifications of confirmed COVID-19 cases were also collected nationally for India from the covid19india website and the Ministry of Health and Family Welfare (MOHFW) website [15,16].

### 2.7. Analysis

Data were extracted in MS excel and then imported to EpiData Analysis version 2.2.2.186 (EpiData Association, Odense, Denmark) and Stata v13 (Stata Corporation College Station, TX, USA) for further cleaning and analysis. A descriptive analysis was performed with frequencies and proportions. The monthly distribution of confirmed COVID-19 cases for Kerala and India was presented by the date of reporting as epi-curves. The total number of COVID-19 cases over the two-year period, stratified by gender, age group, and district were presented in tables, and the districts were also presented on a map as attack rates per million population. The population for the state in 2020 was estimated using the census population figures of 2011 and the decadal growth rate. The number of COVID-19 deaths reported each month in Kerala was documented and case fatalities were presented in relation to gender, age, and district: they were calculated by dividing the total number of deaths over the time period by the total number of reported COVID-19 cases. Case fatality comparisons between different groups were analyzed using the chi-squared test for trends with levels of significance set at 5% (*p* < 0.05). Average bed occupancy each month was calculated for the three different types of field hospitals and hospitals, including intensive care units and placements on mechanical ventilation.

## 3. Results

### 3.1. COVID-19 Cases and the Public Health Responses in Kerala and India

**Figure 1:**
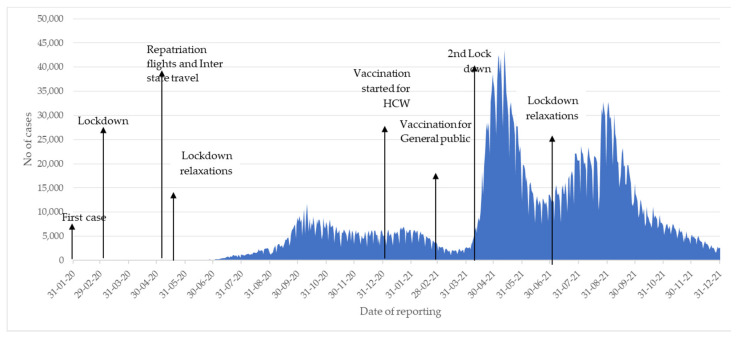
Monthly number of cases of COVID-19 in Kerala, 2020–2021.

**Figure 2:**
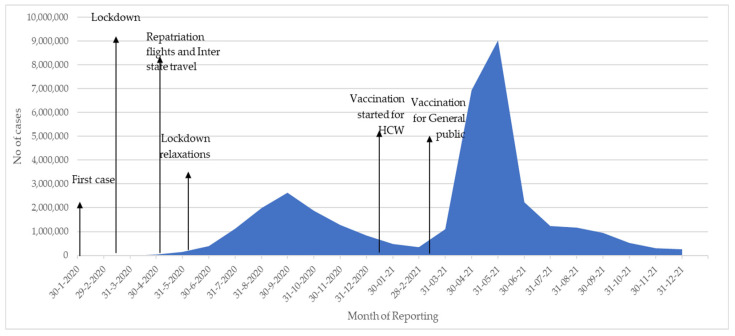
Monthly number of cases of COVID-19 in India, 2020–2021.

The monthly number of new reported cases of COVID-19 from January 2020 to December 2021 in Kerala and in India are shown in Figure 1 and Figure 2 respectively.

The first case of COVID-19 was reported in Kerala (and in India) in January 2020. In Kerala, there were few reported cases in the first 6 months of 2020, following which there were 3 main phases. The first phase started in June 2020 and lasted 6 months with the highest monthly peak reaching 236,999 cases in October 2020. The second phase started in April 2021 with the highest monthly peak reaching 955,396 in May 2021. The third phase started in July 2021 with the highest monthly peak reaching 666,472 in August 2021 and with numbers declining drastically in November and December.

In India, there were also few reported cases in the first 6 months of 2020 following which there were just two main phases. The first phase started in June 2020 and lasted 6 months with the highest monthly peak reaching 2,622,323 in September 2020. The second phase started in April 2021 with the highest monthly peak reaching 9,016,687 in May 2021. Similar to Kerala, numbers declined drastically in November and December.

In terms of response, the timing of national and state lockdowns, the lifting of lockdown restrictions, and the initiation of COVID-19 vaccination are also shown in Figure 1 and Figure 2. The first national lockdown on 24 March 2020 occurred at the same time in Kerala and India. Enforcement of universal usage of facemasks and implementation of the “Break the Chain” campaign to increase awareness amongst the public about the most appropriate social behavior were also initiated in Kerala during March and April. The second lockdown in Kerala occurred in May 2021 and this was different from India where different states enforced lockdown at different times. The initiation of COVID-19 vaccination occurred on 16 January 2021 in Kerala and India. Two vaccines were used. The Oxford-Astra Zeneca COVID-19 viral vector vaccine (Covishield) and the whole inactivated virus-based vaccine developed by Bharat Biotech BBV 152 (Covaxin). In response to the third phase in Kerala, area-specific lockdowns occurred in relation to test positivity rates and reported district incidence of the disease.

### 3.2. Demographic Characteristics and Geographical Distribution of COVID-19 in Kerala

Demographic characteristics and geographical distribution of patients notified with confirmed COVID-19 over the whole two-year period in Kerala from 2020 to 2021 are shown in Table 1.

There were slightly more males than females. The most common age group affected was between 20–40 years, with 3% of children aged up to 5 years and 16% of adults over 60 years also affected. There were five districts (Ernakulam, Malappuram, Kozhikode, Thrissur, and Thiruvananthapuram) that each reported over 500,000 COVID-19 cases, comprising between them over half (54%) of the total number of cases in the state.

**Figure 3:**
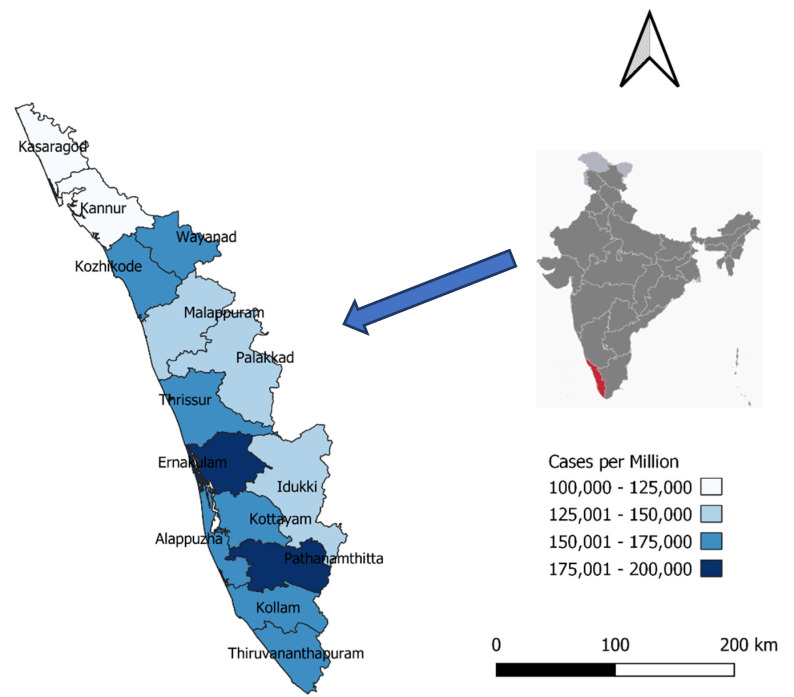
COVID-19 cases per million in the 14 districts of Kerala over the two-year period, 2020–2021.

As each district varied in size of the population, a better measure of the COVID burden, namely the COVID-19 cases per million population over the two years, is shown in Figure 3. During these two years, attack rates per million exceeded 160,000 in five districts (Ernakulam-187,096, Pathanamthitta-17,966, Kottayam-173,082, Kozhikode-170,232, and Thrissur-168,807) and were lowest in the two districts of Kannur (111,166) and Kasaragod (102,049).

### 3.3. COVID-19 Deaths and Case Fatalities

**Figure 4:**
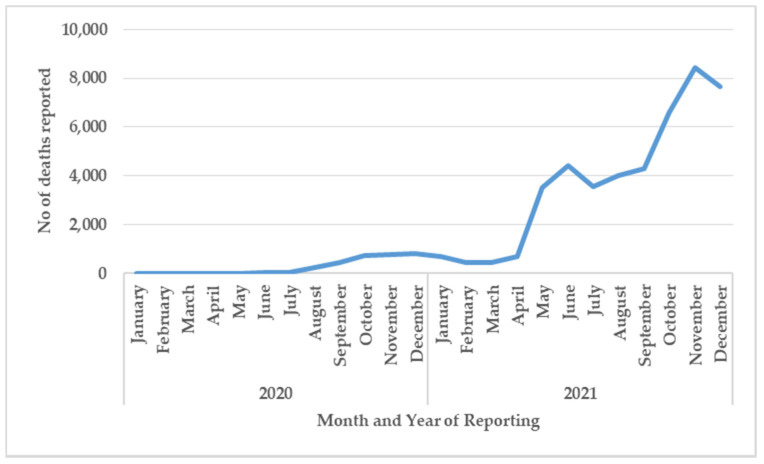
Number of monthly reported deaths due to COVID-19 in Kerala, 2020–2021.

The number of reported COVID-19 deaths each month in Kerala between 2020–2021 is shown in Figure 4.

After June 2021, death reporting changed from a manual to an online system. After September 2021, deaths were defined as occurring within 30 days of COVID-19 test positivity. After October 2021 families could also appeal to have missing COVID-19 deaths included in the registers.

The number of monthly deaths remained low for the first six months and gradually increased to less than 1000 deaths per month up to April 2021. Deaths then increased in May, June, and July to around 3,000-4,000 deaths per month and then increased again in September to reach a peak of over 8,000 deaths in November.

The total number of COVID-19 cases, deaths, and case fatalities for the two-year period in relation to gender, age, and district are shown in Table 2.

The overall case fatality in Kerala during the two-year period was 0.91%. The case fatality in males was significantly higher than in females (1.19% versus 0.70%, *p* < 0.001). There was a significant linear increase in case fatality with each 10-year age group, starting at 0.03% for children aged 0–10 years, rising to 1.15% in adults aged 51–60, and peaking at 10.79% for elderly adults aged over 80 years (*p* < 0.001, chi-squared test for trend). There were variations between districts with five districts (Thiruvananthapuram, Kannur, Kollam, Alappuzha, and Palakkad) having case fatalities above 1%, which were significantly higher than three districts (Malappuram, Idukki, and Wayanad) with case fatalities below 0.7% (*p* < 0.001).

### 3.4. Hospitalization

**Figure 5:**
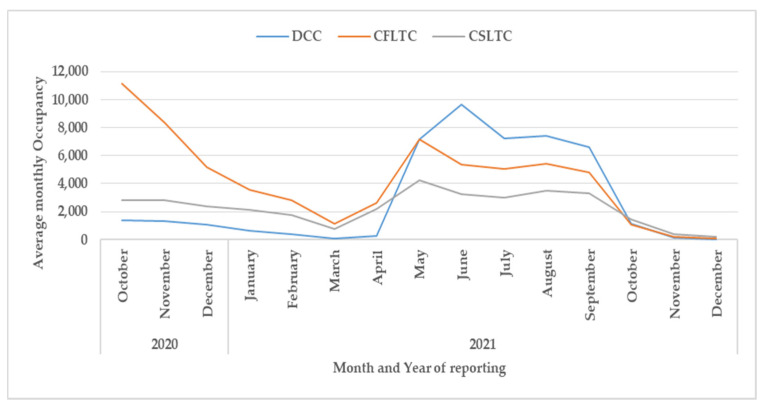
Monthly bed occupancy numbers for the three different types of field hospitals in Kerala, October 2020 to December 2021. DCC = Domiciliary Care Center: for treatment of asymptomatic patients (asymptomatic COVID-19 patients were also permitted home treatment if there were adequate facilities for isolation at home). CFLTC = COVID First-Line Treatment Center: for treatment of patients with minor symptoms. CSLTC = COVID Second Line Treatment Center: for treatment of patients with moderate symptoms.

**Figure 6:**
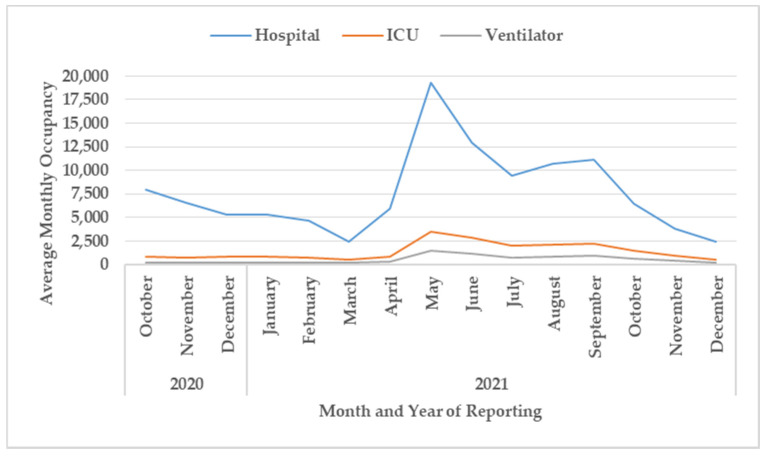
Monthly bed occupancy for hospitals treating severe cases of COVID-19, intensive care units, and mechanical ventilation in Kerala, October 2020 to December 2021. ICU = intensive care unit; Ventilator = mechanical ventilation.

Monthly bed occupancy in relation to the different types of health care facilities is shown in Figure 5 and Figure 6.

For all three field hospitals—Domiciliary Care Centers (DCC), COVID First-line Treatment Centers (CFLTC), and COVID Second Line Treatment Centers (CSLTC)—(Figure 5), the bed occupancy was high in October 2020, gradually declined up to March 2021, then increased again to a peak in June 2021 and then declined dramatically from October 2021 onwards. In the first six months, CFLTC had the highest bed occupancy while from May to October 2021 DCC had the highest bed occupancy.

For hospitals treating severe diseases (Figure 6), the bed occupancy gradually declined between October 2020 and March 2021, increasing dramatically to a peak of nearly 20,000 in May 2021 with a gradual and then a steeper decline thereafter. Bed occupancy in intensive care units and for mechanical ventilation mirrored the trend in hospital bed occupancy.

## 4. Discussion

Using data from the daily COVID-19 bulletins published on the website of the Department of Health and Family Welfare, Kerala, there were three key findings of interest.

First, the epidemiology of monthly cases in Kerala was broadly similar to what was being reported in India, except for a notable third phase of cases in Kerala in August 2021 and slight temporal differences in the timing of events and response measures. The difference in response measures at the national level is not surprising as the various states in India after the first national lockdown had the authority to institute and implement measures as and when they thought necessary, resulting in the average picture being different from that seen in individual states.

The possible reasons for the third phase in Kerala may include the following. In the second phase with state-wide lockdown, COVID-19 cases started declining very rapidly. As a result, lockdown restrictions were eased, the mobility of people increased, and cases started to increase again. This time coincided with the pre-festival and festival season which resulted in the close mingling of large numbers of people. A seroprevalence study among the population in Kerala earlier in the year showed only 44% of the population had antibodies to the virus, contrasting with the 68% found in India as a whole [17]. The relative low immunity in the Kerala population might have also explained their vulnerability to the surge in new infections.

In terms of demographics, the gender and age profile of cases in Kerala were in line with the national profile except for a slight excess of COVID-19 in persons aged 60 years and above (8.6% of people in India were aged more than 60 years compared to 12.6% in Kerala at the time of the national census in 2011 [18]). Within Kerala, some districts had much higher attack rates than others. The reasons are unclear but may be due to different densities of district populations, some districts sharing borders with neighboring states causing seamless inter-border travel for jobs, business as well as for COVID-19 care, different adherence to lockdown restrictions, and different degrees of vaccination uptake. Exploratory qualitative research would be needed to find answers to this question.

Second, from March 2021 onwards, and coinciding with the last two phases of COVID-19 cases in the state, there was a large increase in the number of COVID-19 deaths. How far these increased deaths were due to increased case numbers is difficult to discern. This is because of the system changing from manual to online reporting of deaths in June 2021, a change in the case definition of a COVID-19 death in September 2021, and the retrospective inclusion of past COVID-19 deaths resulting from a Supreme Court of India decision in October 2021 [12].

India has not been alone in changing the way it reports on COVID-19 deaths. In the UK, for example, Public Health England changed its definition of death in mid-2020 leading to an under-reporting of deaths by nearly 75% [19]. In Kerala, the changes may have had the opposite effect resulting in the over-reporting of deaths compared to the previous time period. Given these inaccuracies, there has been a call for countries and states to also report on “excess deaths”, that is the number of all deaths during the pandemic compared with what would have been expected if there had been no pandemic [20,21]. This would allow deaths directly due to the virus to be counted as well as indirect deaths due to patients with non-communicable diseases, especially cancer, not being able to access health services. Experts believe this would be a better measure of the impact of the pandemic on the burden of death in communities and countries.

Despite these concerns about the reporting of deaths, there was a strong association in Kerala between male gender and increasing age with increased case fatality. These associations have been described since the start of the pandemic in low-income, middle-income, and high-income countries [22,23,24,25]. Although we did not document comorbidities in this study, India has the second-highest prevalence of diabetes mellitus in the world after China [26], and in Kerala, it is estimated that 20–25% of the adult population is living with this disease [27]. Data suggest that persons with diabetes mellitus have an increased risk of being infected with COVID-19 [28], and those with diabetes are at much higher risk of severe disease requiring hospitalization, multi-organ failure, coagulopathy, and death compared with those who do not have DM [29,30]. These associations between diabetes (which occurs in older age groups) and COVID-19 may also partially explain the increased mortality rates seen in those of increasing age.

Third, the overall trends in bed occupancy in the different health facilities from the domiciliary care centers to hospitals mirrored the different phases of the pandemic in Kerala. The one difference with the field hospitals was the preponderance of COVID-first-line treatment centers in late 2020 which then changed to a preponderance of domiciliary care centers in 2021. We do not have precise reasons for this change, but it was possibly due to a disproportionate increase in cases, more asymptomatic patients without facilities for home isolation, less severe variants appearing in the population, and the effects of vaccination causing less severe disease in those who had been vaccinated. The beds required for intensive cases and mechanical ventilation also changed in line with the overall trends in hospital bed occupancy. However, in the future, as more people become double or triple vaccinated with better protection against serious illness and death [31,32], the demand for intensive care and mechanical ventilation may drastically decline.

The strengths of this study were the reliable and accurate daily COVID-19 bulletins on the state of the pandemic in Kerala, the large numbers of patients on which to provide estimates, and the conduct and reporting of the study according to the STROBE guidelines (Strengthening the Reporting of Observational Studies in Epidemiology) statement [33].

There were, however, some limitations. First, it would have been helpful to have more data on other variables such as socio-economic characteristics and associated co-morbidities, especially diabetes, of patients notified with COVID-19. Second, we did not have information in the database about how the laboratory confirmed COVID-19, whether by molecular diagnostics or by rapid diagnostic antigen testing. Third, the changing case definitions of COVID-19 deaths also made it difficult to know whether the magnitude of increase of COVID-19 deaths in the latter half of 2021 was real and related to the increase in cases or a feature of the reporting system. In particular, the change which occurred in October 2021 that allowed missing COVID-19 deaths to be retrospectively included in the registers, might have increased the number of reported deaths from that time onwards. We were unable to control these changes and had to just document what had been reported in the routine surveillance system. Fourth, we did not have information on case fatality for the different types of hospitals or about bed occupancy rates, and this would have been useful to better understand care and treatment issues. Finally, for this study, we collected no data on the SARS-CoV-2 variants which occurred in India or Kerala during this time period. Once viral variants began to be reported from other parts of the world, India set up the Indian SARS-CoV-2 Genomics Consortia (INSACOG) and over the two years (2020–2022) identified six variants of concern: Alpha, Beta, Gamma, Delta, B.1.617.1 and B.1.617.3, AY series. A three-prong surveillance strategy was set up focused on international travelers, routine sentinel surveillance, and surge surveillance. However, reporting data on these variants, their arrival in the community, and their impact on cases, hospitalizations and deaths were outside the scope of our study and would require a separate study on its own.

Despite these limitations, there are a number of important implications of these findings. First, the collection of data from the daily COVID-19 bulletins allowed the pandemic in Kerala to be mapped and better interpreted over the two-year period. This strongly suggests that financial and human resource support for this monitoring activity needs to be continued. Bed occupancy at the various hospitals and for intensive care and mechanical ventilation is especially useful for health service planning. The monitoring activities would also benefit from being strengthened with the addition of other variables so that the pandemic can be better understood.

Second, Kerala should consider additional reporting on “excess deaths” as was suggested earlier [20,21]. The World Health Organization and the United Nations Department of Economic and Health Affairs have already established a technical advisory group to estimate the global burden of excess mortality due to COVID-19 [34]. The Ministry of Health in Kerala could liaise with this group about how to calculate this additional metric.

Finally, more research should be undertaken to explain why there are differences in COVID-19 case notifications and case fatalities between the 14 districts of Kerala, and whether these relate to socio-demographic characteristics, access to health services, quality of health service delivery, and especially uptake of COVID-19 vaccination.

## 5. Conclusions

Using data from daily COVID-19 bulletins, the epidemiological characteristics of the COVID-19 pandemic in Kerala were mapped between January 2020 and December 2021. This allowed trends in COVID-19 cases and deaths to be ascertained, case fatality assessed in relation to gender, age, and district, and bed occupancy to be plotted in relation to various levels of hospital care, including intensive care and mechanical ventilation. The routine monitoring system in Kerala has allowed certain aspects of the pandemic to be mapped, but it would benefit from further support and strengthening to increase its public health value.

## Figures and Tables

**Table 1:** Demographic characteristics and demographic distribution of COVID-19 patients in Kerala, 2020–2021.

Characteristic	Category	*n*	(%)
Total cases		5,247,177	
Gender			
	Female	2,422,378	(46.1)
	Male	2,708,870	(51.7)
	Transgender	115,929	(2.2)
Age group in years			
	0–5	157,628	(3.0)
	6–20	772,793	(14.7)
	21–40	1,824,510	(34.8)
	41–60	1,557,464	(29.7)
	>60	818,853	(15.6)
Districts			
	Ernakulam	645,467	(12.3)
	Malappuram	578,930	(11)
	Kozhikode	559,458	(10.7)
	Thrissur	550,280	(10.5)
	Thiruvananthapuram	508,240	(9.7)
	Kollam	412,782	(7.9)
	Palakkad	384,038	(7.3)
	Kottayam	345,063	(6.6)
	Alappuzha	326,260	(6.2)
	Kannur	292,502	(5.6)
	Pathanamthitta	206,322	(3.9)
	Idukki	158,616	(3)
	Kasaragod	143,703	(2.7)
	Wayanad	135,516	(2.6)

**Table 2:** Total COVID-19 cases, total deaths, and case fatalities in Kerala, 2020–2021.

Category	Variable	Total Cases	Total Deaths	Case Fatality %
Total cases		5,247,177	47,794	0.91
Gender				
	Female	2,708,870	19,031	0.70
	Male	2,422,378	28,763	1.19
Age group in years				
	0–10	412,422	114	0.03
	11–20	628,254	80	0.01
	21–30	943,771	354	0.04
	31–40	946,504	1309	0.14
	41–50	853,776	3595	0.42
	51–60	708,032	8127	1.15
	61–70	469,737	13,022	2.77
	71–80	204,886	12,583	6.14
	>80	79,795	8610	10.79
Districts				
	Thiruvananthapuram	508,240	6274	1.23
	Kannur	292,502	3493	1.19
	Kollam	412,782	4576	1.11
	Alappuzha	326,260	3600	1.1
	Palakkad	384,038	3946	1.03
	Thrissur	550,280	5303	0.96
	Pathanamthitta	206,322	1820	0.88
	Ernakulam	645,467	5560	0.86
	Kottayam	345,063	2676	0.78
	Kozhikode	559,458	4274	0.76
	Kasaragod	143,703	922	0.64
	Malappuram	578,930	3670	0.63
	Idukki	158,616	975	0.61
	Wayanad	135,516	705	0.52

Case fatality is calculated by dividing deaths by cases.

## Data Availability

The data for this paper is available upon request from the principal investigator.

## Open Access Statement and Disclaimer

In accordance with WHO’s open-access publication policy for all work funded by WHO or authored/co-authored by WHO staff members, WHO retains the copyright of this publication through a Creative Commons Attribution IGO License (http://creativecommons.org/licenses/by/3.0/igo/legalcode) which permits unrestricted use, distribution and reproduction in any medium provided the original work is properly cited. There should be no suggestion that WHO endorses any specific organization, products, or services. The views expressed in this article are those of the authors and do not necessarily reflect those of their affiliated institutions. The use of the WHO logo is not permitted. This notice should be preserved along with the article’s original URL.
